# Glucose-dependent inflammatory responses in obese compared to lean individuals

**DOI:** 10.1007/s12020-023-03433-4

**Published:** 2023-07-03

**Authors:** Martin H. Lundqvist, Maria J. Pereira, Jan W. Eriksson

**Affiliations:** Clinical Diabetology and Metabolism, Department of Medical Sciences, Uppsala University, Uppsala, Sweden

**Keywords:** Inflammation, Obesity, Insulin resistance, Hypoglycemia, Hyperglycemia

## Abstract

**Purpose:**

Obesity is characterized by chronic inflammation that may contribute to insulin resistance and promote type 2 diabetes. We have investigated whether inflammatory responses to glycemic and insulinemic variations are altered in obese individuals.

**Methods:**

Eight obese and eight lean individuals without diabetes had undergone hyperinsulinemic-euglycemic-hypoglycemic and hyperglycemic clamps in a previous study. Using Proximity Extension Assay, 92 inflammatory markers were analyzed from plasma samples at fasting, hyperinsulinemia-euglycemia, hypoglycemia and hyperglycemia.

**Results:**

In all participants, hyperinsulinemia, hypoglycemia and hyperglycemia led to reductions of 11, 19 and 62 out of the 70 fully evaluable biomarkers, respectively. FGF-21 increased during both hypoglycemia and hyperglycemia while IL-6 and IL-10 increased during hypoglycemia. In obese vs lean participants, Oncostatin-M, Caspase-8 and 4E-BP1 were more markedly suppressed during hypoglycemia, whereas VEGF-A was more markedly suppressed during hyperglycemia. BMI correlated inversely with changes of PD-L1 and CD40 during hyperinsulinemia, Oncostatin-M, TNFSF14, FGF-21 and 4EBP-1 during hypoglycemia and CCL23, VEGF-A and CDCP1 during hyperglycemia (Rho ≤ -0.50). HbA1c correlated positively with changes of MCP-2 and IL-15-RA during hyperinsulinemia (Rho ≥ 0.51) and inversely with changes of CXCL1, MMP-1 and Axin-1 during hypoglycemia (Rho ≤ -0.55). M-value correlated positively with changes of IL-12B and VEGF-A during hyperglycemia (Rho ≥ 0.51). Results above were significant (*p* < 0.05).

**Conclusion:**

Overall, hyperinsulinemia, hypo- and hyperglycemia led to suppression of several inflammatory markers and this tended to be more marked in individuals with obesity, insulin resistance and dysglycemia. Thus, acute glycemic or insulinemic variations do not seem to potentiate possible inflammatory pathways in the development of insulin resistance and disturbed glucose metabolism.

## Introduction

The association between type 2 diabetes and overweight is well established; 80–90% of patients with type 2 diabetes are overweight or obese [1, 2] and obesity is one of the strongest modifiable risk factors for developing type 2 diabetes [3]. The exact mechanisms by which obesity induces insulin resistance and ultimately type 2 diabetes are incompletely understood but obesity-induced low-grade inflammation has been suggested as a putative link [4].

In obesity, macrophages infiltrate the adipose tissue and polarize to the proinflammatory phenotype M1 that preferentially secretes proinflammatory cytokines such as TNF-α, IL-6 and IL-8 [5, 6]. Proinflammatory cytokines are increased in obesity and type 2 diabetes, both in the circulation and in the adipose tissue [7–10], whereas some anti-inflammatory cytokines, such as adiponectin, are reduced in these conditions [11, 12]. Following bariatric surgery-induced weight loss, adipose tissue gene expression of some proinflammatory cytokines is slightly reduced [13], while circulating levels remain unchanged [10], highlighting the chronic nature of this inflammatory state. Apart from promoting inflammation, these cytokines have also been shown to exert unfavorable systemic metabolic effects by, for instance, impairing insulin signaling in insulin-sensitive tissues as well as insulin secretion from beta cells [14, 15]. Other inflammatory markers, such as FGF-21 and VEGF-A have been demonstrated to have favorable metabolic properties [16, 17], while IL-6 may exert both detrimental and favorable metabolic effects depending on the context and organ-site of action [18]. Thus, the link between obesity, inflammation and insulin resistance is complex and, in many aspects, incompletely understood.

Most clinical studies addressing the role of inflammatory mediators in metabolic diseases have been performed with participants under fasting conditions at their ambient glucose levels. However, rapid fluctuations of glucose and insulin levels may also modulate the degree of inflammatory activity, of particular relevance in prediabetes and diabetes. Hypoglycemia leads to a physiological response not only of counter-regulatory hormones such as glucagon, cortisol, catecholamines and growth hormone, but also of cytokines such as TNF-α, IL-6, IL-8 and VEGF-A [19–21]. Interestingly, both TNF-α and IL-6 have been shown to increase similarly during experimental hyperglycemia along with rises in IL-18 and FGF-21 [22–25] and suppression of VEGF [26]. Insulin have been demonstrated to suppress many inflammatory markers [23, 27, 28] and to stimulate the polarization of macrophages to the anti-inflammatory M2 phenotype [29]. In contrast to the well-characterized low-grade chronic inflammation associated with obesity, less is known about dynamic inflammatory responses to such short-term metabolic fluctuations in obesity and insulin resistance. In individuals with type 2 diabetes, augmented hypoglycemic responses of various inflammatory markers, including VEGF, and an attenuated rise of IL-6 during hyperinsulinemia have been reported [30–32]. Similarly, augmented hyperglycemic responses of TNF-α, IL-6 and IL-18 have been observed in individuals with prediabetes [22].

Previously, using glucose clamp techniques, our research group has demonstrated that overweight and insulin resistant individuals have augmented responses of the cortisol axis to hypoglycemia and impaired suppression of glucagon during hyperglycemia [33]. In the present study, we have used EDTA plasma samples from that study to determine the inflammatory responses to hyperinsulinemia, hypoglycemia and hyperglycemia in obese compared to lean individuals. We hypothesized that these responses would be altered in obese compared to lean individuals, thereby possibly acting in concert with the observed glucose-dependent hormonal disturbances to promote development of insulin resistance and ultimately type 2 diabetes in these individuals.

## Methods

### Participants

The experiments in this study were conducted at Uppsala University Hospital and at Uppsala University between March 2018 and October 2019. Individuals with no previous diagnosis of diabetes, BMI 18.5–50 kg/m^2^ and age 18–60 years were included. Exclusion criteria were other diseases or medication that could influence the outcome or the individual’s ability to comply with the study procedures, planned or ongoing pregnancy and significant substance abuse. The study procedures will be described briefly below, but for details we refer to the original publication [33]. For this study, we analyzed inflammatory markers in stored samples from eight lean and eight obese individuals with no history of acute or chronic inflammatory or infectious disease.

### Study design

The protocol details are previously reported [33]. We provide a brief description here. The participants arrived at the unit on two occasions separated by 1–5 weeks, around 8.00–9.00 AM after an overnight fast of at least 10 h. In a randomized order, stepwise hyperinsulinemic-euglycemic-hypoglycemic clamps and hyperglycemic clamps were performed at these visits. Anthropometrics, body composition using bioimpedance (Tanita body composition analyzer, BC-418; Tanita corporation. Tokyo. Japan) and fasting blood samples were obtained before the clamp start at approximately 9 AM.

The hyperinsulinemic-euglycemic-hypoglycemic clamp (henceforth referred to as hypoglycemic clamp) started with simultaneous infusions of insulin (56 U/m2 body surface area after priming), potassium chloride (8 mmol/h) and glucose 200 mg/ml. The glucose infusion rate was varied to maintain blood glucose levels at 5 mmol/l for the first 80 min (hyperinsulinemic-euglycemic phase) after which blood glucose was lowered stepwise to 3.8 mmol/l (for 30 min), 3.2 mmol/l (for 45 min) and 2.7 mmol/l (for 30 min). The insulin infusion was then terminated and glucose was infused at a fixed rate of 200 mg/kg/h or 300 mg/kg fat-free mass (FFM)/h until glucose levels had returned to > 5 mmol/l. M-value was defined as the mean glucose infusion rate from 40 to 80 min during the hyperinsulinemic-euglycemic phase.

The hyperglycemic clamp started with a variable infusion of glucose to maintain glucose levels at the basal, fasting level for 30 min., after which the infusion rate was adjusted to achieve stepwise glucose plateaus of 3 mmol/l, 6 mmol/l and 9 mmol/l above the fasting levels for 45 min each. Thereafter, the glucose infusion was terminated to allow glucose levels to return to the normal range.

**Fig. 1:**
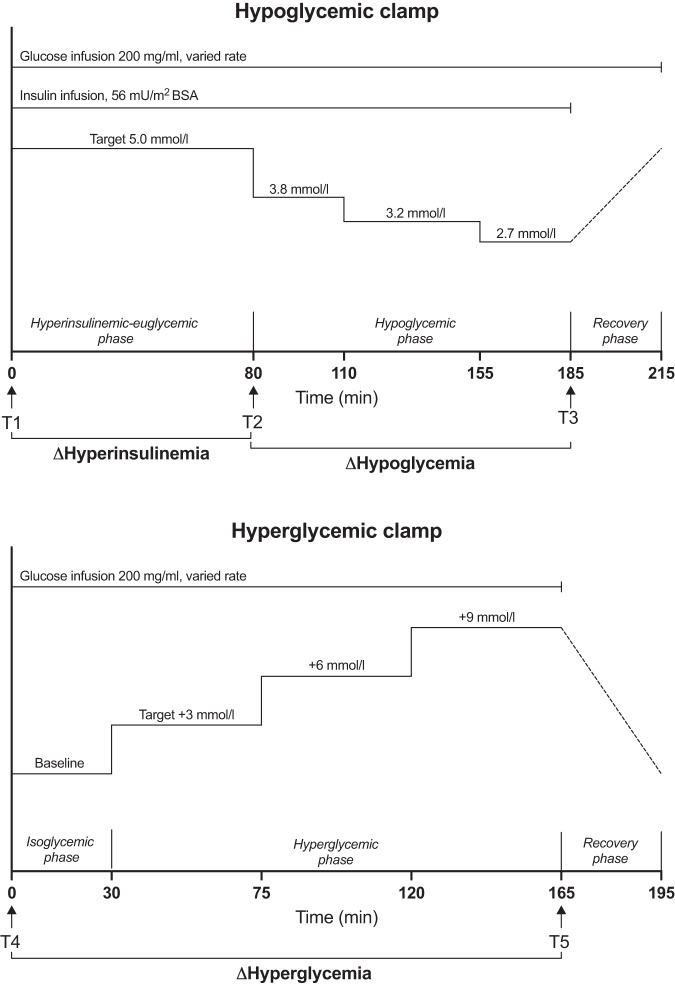
Design of hypo- and hyperglycemic clamps with indicated timepoints (T1-T5) where samples of inflammatory markers were obtained and indicated intervals for Δ-values. BSA Body surface area

Throughout the clamps, glucose was measured from blood taken from an arterialized vein using a Contour glucometer (Bayer Healthcare, Leverkusen, Germany). Blood samples for hormonal analyses were obtained from the same arterialized vein every 15–30 min of the clamps. After centrifugation, EDTA-plasma was frozen and stored at −80 °C in the biobank at Uppsala University Hospital (number 827). For this study, biobank samples from five timepoints were thawed and analyzed: T1) fasting before the hypoglycemic clamp, T2) at 80 min of hypoglycemic clamp (hyperinsulinemia and euglycemia), T3) at 185 min of hypoglycemic clamp (end of hypoglycemic phase, target glucose level 2.7 mmol/l), T4) fasting before the hyperglycemic clamp and T5) at 165 min of hyperglycemic clamp (end of hyperglycemic phase, target glucose 9 mmol/L above fasting level). Figure. 1 illustrates the clamp designs and relevant timepoints.

### Biochemistry

Inflammatory markers were measured from EDTA-plasma using Proximity Extension Assay technology provided by Olink® proteomics (Watertown. MA. USA). This technique uses DNA-labeled antibodies and DNA sequence-specific protein-to-DNA conversion to generate a signal that is read by quantitative real-time PCR (qPCR). Cross-reactivity issues with traditional immunoassays are thus circumvented and this allows for high-throughput, highly sensitive and specific multiplex protein analyses [34]. The Target 96 Inflammation Panel was used. All 92 inflammatory markers included in this panel are specified in Supplement S1. For more information, visit www.olink.com/downloads. The blood samples in this study were analyzed in two different plates, using bridge sample normalization to allow comparisons. Inflammatory markers that were under the limit of detection (LOD) in more than 25% of the samples in any plate were analyzed separately. Individual values under the LOD were not transformed.

Insulin, cortisol, C-peptide (CobasE, Roche), ACTH and growth hormone (Immulite 2000XPi, Siemens Healthcare Global) were analyzed at the Department of Clinical Chemistry, Uppsala University Hospital. Glucagon was measured with ELISA (#10-1271-01, Mercodia, Uppsala, Sweden, within-assay CV 2.1–14%, between-assay CV 7.0–16%) at the Clinical Diabetes Research Laboratory. Values below the lower limit of quantification (LLQ) for each analyte were imputed to LLQ/2.

### Statistical analysis

Since this was an exploratory study of existing material, no formal power calculation was made. Group differences in clinical characteristics were analyzed with Fisher’s exact test or Mann-Whitney U test. The main outcomes in this study were the absolute change during hyperinsulinemia (ΔHyperinsulinemia, between T1 and T2, Fig. 1), hypoglycemia (ΔHypoglycemia, between T2 and T3, Fig. 1) and hyperglycemia (ΔHyperglycemia, between T4 and T5, Fig. 1) between obese and lean individuals. Olink® inflammatory markers are reported in Normalized Protein eXpression (NPX), which is an arbitrary, dimensionless unit on the log2 scale. Thus, the absolute difference in NPX between two timepoints reflects the fold change. Group differences of fasting levels, ΔHyperinsulinemia, ΔHypoglycemia and ΔHyperglycemia were compared with Mann-Whitney U tests. Within-subject changes during hyperinsulinemia, hypoglycemia and hyperglycemia were analyzed using one-sample Wilcoxon signed-rank test of ΔHyperinsulinemia, ΔHypoglycemia and ΔHyperglycemia for the whole cohort (*n* = 16). Spearman’s rank correlations were computed to assess relationships between inflammatory responses, clinical characteristics and hormonal responses in the whole cohort (*n* = 16). Two-tailed *p*-values < 0.05 were considered significant.

Statistical analysis was carried out using SPSS for Mac version 28 (IBM corp. Armonk. NY. USA). Figures were constructed in GraphPad Prism version 9.1.0 (GraphPad software. San Diego. CA. USA).

## Results

Clinical characteristics of obese (*n* = 8) and lean (*n* = 8) participants are displayed in Table 1. The groups were well-matched according to sex and age. As expected, the obese participants were more insulin-resistant, had higher fasting glucose levels, less favorable lipid profile and higher CRP and Leukocyte Blood Count. One obese female participant had two fasting glucose levels above 7.0 mmol/l but an HbA1c of 40 mmol/mol. This participant was included in the current analyses although she was later diagnosed with type 2 diabetes. Group differences in clamp measurements of glucose, glucose infusion rates and hormones were in accordance with the original study [33] and will not be presented here.

**Table 1:** Biometric and biochemical data obtained at first available occasion

Variable	Lean (*n* = 8)	Obese (*n* = 8)	*p*
Age, years	47 (28;56)	46 (25;55)	0.959
Sex, M/F	2/6	1/7	1.000
BMI, kg/m^2^	22.9 (20.8;24.4)	37.6 (31.0;47.6)	**<** **0.001**
Body fat, %	25.0 (15.9;30.5)	44.3 (29.2;55.2)	**<** **0.001**
Waist/hip ratio	0.83 (0.72;0.97)	0.96 (0.89;1.04)	**0.005**
Systolic BP, mmHg	115 (102;138)	129 (120;148)	**0.050**
Diastolic BP, mmHg	79 (62;88)	88 (76;95)	**0.050**
HbA1c (IFCC), mmol/mol	34 (29;37)	36 (31;41)	0.195
HbA1c (NGSP), %	5.3 (4.8;5.5)	5.4 (5.0;5.9)	0.195
Fasting plasma glucose, mmol/l	5.6 (4.9;6.1)	6.0 (5.4;7.5)	**0.038**
Serum C-peptide, nmol/l	0.6 (0.3;0.8)	1.1 (0.6;1.5)	**<** **0.001**
Serum insulin, pmol/l	29.2 (6.3;61.1)	104.9 (38.2;229.2)	**<** **0.001**
HOMA-IR	1.0 (0.2;2.4)	4.3 (1.4;11.0)	**<** **0.001**
M-value, mg/kg FFM/min	14.0 (7.0;16.7)	7.4 (3.2;11.9)	**0.005**
Plasma CRP, mg/l	1.2 (0.2;1.6)	3.9 (0.6;5.0)	**0.005**
Blood Leukocyte Count, x10^9^	4.6 (3.7;5.3)	5.4 (4.8;7.0)	**0.001**
Plasma Cholesterol, mmol/l	4.6 (3.6;5.1)	4.3 (3.1;5.3)	0.931
Plasma LDL cholesterol, mmol/l	2.5 (1.6;3.3)	3.0 (1.9;3.7)	**0.050**
Plasma HDL cholesterol, mmol/l	1.4 (1.0;2.4)	0.9 (0.8;1.4)	**0.005**
Plasma triacylglycerols, mmol/l	0.65 (0.37;1.23)	0.92 (0.58;2.35)	**0.028**

Data are presented as median (min;max). *BP* Blood pressure, *FFM* Fat-free mass *P*-values refer to comparisons between obese and lean participants with Fischer’s exact test for sex and Mann-Whitney U tests for all other variables.

Bold values indicates statistical significant *P* values.

Out of all 92 inflammatory biomarkers (listed in Supplement S1 with abbreviations explained), 22 were under LOD in at least 25% in any plate. These biomarkers did not differ from the others in terms of between-group differences or interindividual dynamic behavior but are presented separately in Supplement S2.

### Group comparison of fasting levels and dynamic responses of inflammatory markers in obese compared to lean participants

**Fig. 2:**
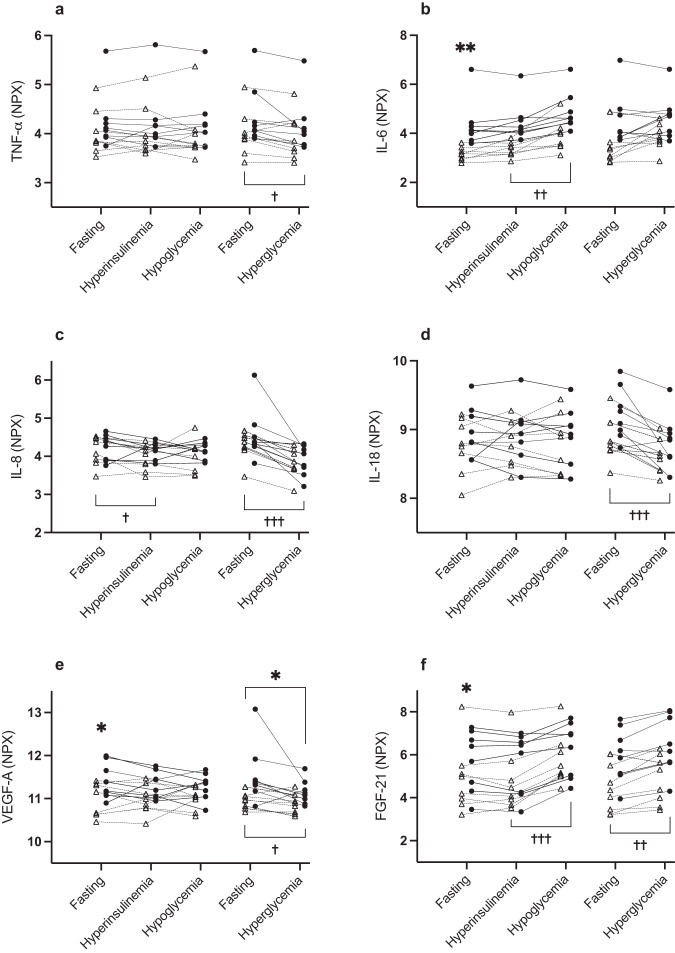
Spaghetti plot of individual levels of main inflammatory biomarkers in obese (filled black circles and solid lines) and lean participants (open triangles and dashed lines) in hypoglycemic (first three timepoints from left) and hyperglycemic clamp (last two timepoints from left). Unit is NPX (Normalized Protein eXpression), logtransformed to base 2. *p < 0.05, **p < 0.01, group comparison by Mann-Whitney U tests of fasting levels (mean of both visits) or Δ-value between indicated timepoints. ^†^*p* < 0.05, ^††^*p* < 0.01, ^†††^*p* < 0.001, one sample Wilcoxon signed-rank tests of Δ-value between indicated timepoints

Results are presented in Table 2. Individual levels of TNF-α, IL-6, IL-8, IL-18, FGF-21 and VEGF-A at different conditions are displayed in Fig. 2. These biomarkers were of specific interest due to previous evidence of dynamic changes to glucose variations and associations with obesity and metabolic disorders.

**Table 2:** Fasting levels and changes of levels (Δ) of inflammatory biomarkers during hyperinsulinemia, hypoglycemia and hyperglycemia

Data presented as median(range).*p < 0.05, **p < 0.01, Mann-Whitney U tests of obese vs lean participants. ^†^*p* < 0.05, ^††^*p* < 0.01, ^†††^*p* < 0.001, one sample Wilcoxon signed-rank tests for all participants (*n* = 16).^a^Data missing for one participant in ΔHyperinsulinemia and ΔHypoglycemia. Abbreviations for inflammatory biomarkers are provided in Supplement S1. Results for markers with large prevalence of values under the limit of detection are provided in Supplement S2

Fasting levels (mean of both visits) were significantly higher for 7/70 biomarkers in obese vs lean participants (Table 2), including IL-6 (Fig. 2b) and VEGF-A (Fig. 2e). By contrast, DNER was lower in obese vs lean participants (Table 2).

#### Hyperinsulinemia

In all participants (*n* = 16), hyperinsulinemia alone led to significant reductions in 11/70 inflammatory markers, including IL-8 (Fig. 2c, Table 2). PD-L1 decreased in obese participants and increased in lean participants and the group difference of the Δ-value was significant. Otherwise, none of the responses differed in obese vs lean participants (Table 2).

#### Hypoglycemia

In all participants, hypoglycemia led to significant reductions in 19/70 inflammatory markers from all subcategories (Table 2). By contrast, IL-6 (Fig. 2b), FGF-21 (Fig. 2f) and IL-10 all increased significantly. OSM decreased in obese participants and increased in lean participants with a significant group difference of the Δ-value. 4E-BP1 and CASP-8 decreased in both groups but significantly more markedly in obese vs lean participants.

#### Hyperglycemia

In all participants, hyperglycemia led to significant reductions of 62/70 inflammatory markers across all subcategories (Table 2) including TNF-α (Fig. 2a), IL-8 (Fig. 2c), IL-18 (Fig. 2d) and VEGF-A (Fig. 2e). By contrast, FGF-21 increased significantly (Fig. 2f) and there was a trend for IL-6 (Table 2, Fig. 2b). Obese participants had a more profound suppression of VEGF-A but otherwise, there were no significant group differences in the responses.

### Correlations between inflammatory responses, metabolic parameters and clamp assessments

Spearman’s rank correlations between inflammatory responses and BMI, HbA1c and M-value are presented in Table 3. Correlations with other metabolic parameters and clamp measurements are presented in Supplement S3–S5.

**Table 3:** Spearman’s rank correlation coefficients between inflammatory responses to hyperinsulinemia, hypoglycemia and hyperglycemia and BMI, fasting glucose and M-value (M-val)




M-value is inversely related to insulin resistance. Significant coefficients are typed in **bold**. Heat-map with background in three-color scale where −1=blue, +1=red and 0=white. Correlations with other metabolic markers and hormonal responses are displayed in Supplement S3-S5. ^a^Data missing for one participant during hyperinsulinemia and hypoglycemia

#### Hyperinsulinemia

In general, obesity measures displayed tendencies toward accentuated suppression of several biomarkers (Table 3, Supplement S3). Body fat % had particularly strong correlations, significant for 9/70 markers (Rho −0.72 to −0.50), whereas BMI correlated significantly with CD40 (Rho −0.52) and PD-L1 (Rho −0.50). Contrarily, markers of chronic hyperglycemia displayed tendencies toward attenuated suppression of inflammatory markers (Table 3, Supplement S3). Significant correlations were found for HbA1c vs MCP-2 (Rho 0.55) and IL-15RA (Rho 0.51). Markers of insulin resistance correlated very weakly with inflammatory responses, with coefficients close to zero and in no consistent direction (Table 3, Supplement S3). CRP followed the same pattern as obesity measures, with significant inverse correlations for 3/70 markers, whereas insulin levels at the end of the hyperinsulinemic-euglycemic phase displayed positive weak tendencies with inflammatory responses (Supplement S3).

#### Hypoglycemia

In general, CRP and measures of obesity, chronic hyperglycemia and insulin resistance displayed tendencies toward more marked suppression or attenuated elevation of several inflammatory markers (Table 3, Supplement S4). Notably, this tendency was particularly strong for fasting glucose, which correlated significantly with a greater suppression of 33/70 inflammatory markers (Rho −0.80 to −0.50) and with an attenuated increase of FGF-21 (Rho −0.65, Supplement S4). HbA1c displayed weaker negative tendencies with significant correlations vs CXCL1, MMP-1 and Axin-1 (Rho −0.58 to −0.55), while the correlation with FGF-19 was significantly positive (Rho 0.58, Table 3). There was a similar general negative tendency for correlations with BMI, that was significant for OSM, TNFSF14, FGF-21 and 4E-BP1 (Rho −0.56 to −0.51, Table 3). M-value displayed weaker tendencies in the positive direction, while CRP displayed negative tendencies with inflammatory responses (all non-significant, Supplement S4). The glucagon response and, to a lesser extent, the growth hormone response to hypoglycemia displayed positive trends, whereas the ACTH/cortisol-response displayed negative trends with inflammatory responses (Supplement S4).

#### Hyperglycemia

There was a general tendency for negative correlations, indicative of more marked suppressions, between BMI and inflammatory markers that were significant with regard to CCL23, VEGF-A and CDCP1 (Rho −0.66 to −0.53, Table 3). M-value displayed a positive tendency that was significant for IL-12B (Rho 0.51) and VEGF-A (Rho 0.56, Table 3). HbA1c displayed weaker, non-significant correlations in no consistent direction (Table 3). Additional metabolic parameters displayed similar patterns (Supplement S5). CRP and insulin_AUC_ displayed strong and consistent negative correlations, significant for 6/70 and 11/70 markers, respectively, whereas the response of glucagon and ACTH/cortisol generally correlated in the positive direction, significant for 2/70 markers vs glucagon and 5/70 markers vs both cortisol as well as ACTH (Supplement S5).

## Discussion

In this study, we examined inflammatory responses to rapid glucose and insulin variations in lean and obese individuals. While substantial group differences were not found, obesity and associated metabolic disturbances correlated with accentuated suppressions or attenuated elevations of several inflammatory markers during both hypo- and hyperglycemia. Given the complex interplay between inflammation and whole-body metabolism, the consequences of these findings are somewhat unclear and a net deleterious metabolic impact, assisting in the development and maintenance of insulin resistance and dysglycemia, cannot be excluded. However, it seems more plausible that the metabolic implications of the observed minor alterations are negligible and that these alterations are at least partly subsidiary to parallel metabolic disturbances of superior pathogenic impact.

### General inflammatory responses to hyperinsulinemia, hypoglycemia and hyperglycemia

Intriguingly, all experimental conditions generally led to reductions in circulatory levels of many inflammatory biomarkers. Of particular interest, a very similar response was observed during hypo- compared to hyperglycemia and only biomarkers with anti-inflammatory or, as previously demonstrated, favorable metabolic properties were observed to rise during these conditions. The reduction of inflammatory markers during hyperinsulinemia are largely in congruency with previous studies [23, 27, 28], while a stimulative effect has been observed with regard to FGF-21 [25]. The U-shaped response of IL-6, the rise of FGF-21 during hyperglycemia and the hyperglycemia-induced suppression of VEGF-A are in keeping with previous studies [20, 22–26, 35]. However, there has been no previous report of hypoglycemia-induced elevations of FGF-21 to our knowledge. In other studies, but not in accordance with the current findings, increases during hypoglycemia has also been described for TNF-α, Il-8 and VEGF-A [19–21], while TNF-α and IL-18 have been demonstrated to increase during hyperglycemia [22, 23]. It should be pointed out that, in the present study, both hypoglycemia and hyperglycemia were achieved in the face of exogenous and endogenous hyperinsulinemia, respectively. Thus, isolated effects of glucose and insulin variations cannot be fully disjointed and it is conceivable that the observed general suppression of inflammatory markers is partly attributable to hyperinsulinemia.

### Impact of metabolic phenotype on inflammatory markers and responses

Several inflammatory biomarkers were elevated in obese compared to lean individuals in the fasting state, including IL-6, VEGF-A and FGF-21. This is in accordance with previous studies [7, 36, 37] and increased levels of several other inflammatory markers, that were not significantly higher in this study, such as TNF-α and IL-8, have also been reported [7, 8]. Inflammatory responses during hyperinsulinemia were not consistently different between the groups and correlation analyses yielded conflicting, possibly serendipitous results. During hypo- and hyperglycemia, the observed group differences as well as correlations with measures of obesity, dysglycemia and insulin resistance were generally in the negative direction, indicative of a more marked suppression. In the hypoglycemic clamp, the particularly strong correlations between inflammatory responses and fasting glucose suggest that these findings may partly be explained by a larger relative decrement of glucose levels during the clamp experiment compared to baseline glucose levels. Moreover, we have previously shown that obese and insulin resistant individuals have an augmented cortisol axis response to hypoglycemia [33, 38]. Thus, the observed negative correlations between BMI as well as other metabolic parameters and hypoglycemic inflammatory responses could in part be mediated by non-genomic anti-inflammatory actions of cortisol. This is supported by the general negative tendencies between the cortisol response and hypoglycemic inflammatory responses. During the hyperglycemic clamp, insulin levels were markedly higher in obese participants [33]. This may have partly explained the more pronounced suppression of inflammatory markers during this condition, considering the previously described anti-inflammatory actions of insulin [23, 27, 28] and the strong negative correlations between hyperglycemic clamp insulin levels and inflammatory responses observed in this study. From a different standpoint, it is also possible that a habitually higher inflammatory activity in obese individuals normalizes with respect to lean individuals in the context of near-maximum insulin stimulation and similar glucose deprivation or excess. After all, chronic inflammation in obesity and associated conditions does not seem to be inert to rapid metabolic manipulation, judging by the current results. Importantly, the current sample size did not allow for detailed investigation, for instance in regression models, of the mediating impact of the concomitant metabolic features described above. Moreover, the modulatory impact of free fatty acids, previously demonstrated to stimulate secretion of proinflammatory cytokines [39] and lead to macrophage M1 polarization [40], or other nutrients have not been explored in the current study.

### Interaction between counter-regulatory hormones and inflammatory biomarkers

In both clamps, the glucagon response correlated positively with the glucose-dependent responses of several inflammatory markers. This is compatible with the recently demonstrated pro-inflammatory action of glucagon in the liver [41] although anti-inflammatory effects in other tissues are also supported by previous work [42]. Interestingly, cortisol and ACTH responses were negatively correlated with the inflammatory responses in the hypoglycemic clamp and positively in the hyperglycemic clamp. Since the interplay between inflammation and cortisol is bidirectional, it is possible that the finding in the hypoglycemic clamp reflects the immunosuppressive effect of cortisol whereas the finding in the hyperglycemic clamp reflects the stimulatory effect of inflammatory markers on activation of the cortisol axis [43]. The previously reported association between cortisol responses and the IL-6 response to hypoglycemia [21] could not be confirmed in this study.

### Metabolic implication of current findings

Epidemiologic associations between metabolic alterations and inflammatory markers [7–10]. as well as in vitro evidence of detrimental metabolic effects of some of these markers [14, 15] underly the postulated pathogenetic role of inflammation in the development of type 2 diabetes. However, favorable metabolic effects have been attributed to some inflammatory markers [16–18] and attempts to pharmacologically target inflammation as treatment for type 2 diabetes have been largely disappointing [44]. In mouse models, suppression of adipose tissue inflammation has even led to worsening of systemic insulin resistance [45, 46], indicating that the chronic inflammation that characterizes metabolic disorders may be a protective response from a metabolic standpoint, rather than a causative factor for type 2 diabetes development and maintenance of hyperglycemia. Thus, it may be speculated that inflammatory downregulation during rapid glycemic changes outside the normal range represents a defense response contributing to the physiological counter-regulation aiming towards recovery of normoglycemia.

### Strengths and limitations

In this study, we have performed a comprehensive assessment of a wide panel of inflammatory markers at distinctly defined experimental metabolic conditions and in individuals with a wide range of chronic metabolic phenotypes. Thus, detailed mapping of the impact of various metabolic factors on glucose- and insulin-dependent inflammatory responses has been possible, and new insight into general aspects of these responses has been gained. Previous studies investigating glucose-dependent inflammatory responses have generally assessed a limited number of inflammatory markers and comparison of these responses between obese and lean individuals has not been reported previously, to our knowledge. This study is limited by its small sample size and the resulting restriction of statistical power. Second, due to the co-regulation and interdependency of many inflammatory markers, we did not formally adjust for multiple comparisons in this exploratory study. While there is an ongoing debate regarding the necessity of such multiplicity adjustments [47], we acknowledge the risk of resulting type I errors and recognize the need for confirmation of the current hypothesis-generating findings in a larger cohort. Third and as previously discussed, the current clamp designs did not permit separate estimations of glucose and insulin effects on the outcome variables. Profound hypoglycemia is not achievable in the absence of experimental hyperinsulinemia but the addition of a control experiment with a prolonged hyperinsulinemic-euglycemic phase could potentially have allowed for isolated scrutiny of hypoglycemic effects. However, the protocol was already demanding for the study participants and the study was not primarily designed to address the current research question. Moreover, inference regarding group differences and impact of metabolic parameters on the outcome variables are still valid notwithstanding this limitation. In the hyperglycemic setting, a somatostatin clamp design with controlled infusions of pancreatic hormones and glucose may have provided a more accurate estimation of pure hyperglycemic effects on inflammatory responses. However, this experimental design deviates substantially from normal physiology and somatostatin has anti-inflammatory properties [48] that could have interfered with the results along with its well-known indiscriminate inhibition of several endocrine systems. Finally, the timeframes for the experimental conditions may not have been sufficient to unveil some true within-subject and between-subject differences, possibly explaining minor discrepancies with other studies.

## Conclusion

In this study, we did not find a general pro-inflammatory response to short-term hyperinsulinemia, hypoglycemia or hyperglycemia in lean and obese individuals. Surprisingly, several inflammatory mediators were suppressed during all these conditions and these suppressions tended to be more marked in individuals with obesity, dysglycemia and insulin resistance. Thus, while chronic low-grade activation of inflammatory pathways in obesity may contribute to insulin resistance and the development of type 2 diabetes, acute glycemic or insulinemic variations do not seem to potentiate such perturbations. The current findings are to be considered hypothesis-generating and confirmation in larger cohorts, ideally including individuals with type 2 diabetes, is warranted.

## Supplementary information

Supplementary Information

## Data Availability

Data will be made available upon reasonable request to the corresponding author.
