# Supplementary material for: Glucose-dependent inflammatory responses in obese compared to lean individuals
Source: Endocrine. 2023 Jul 3;81(3):464–76. doi: 10.1007/s12020-023-03433-4 (PMC10403442; doi:10.1007/s12020-023-03433-4)
Supplement: Supplementary file 1 — Supplementary Information [file 12020_2023_3433_MOESM1_ESM.docx]

**Supplement**

**S1. Abbreviations of inflammatory markers**

| Adenosine Deaminase (ADA) | Hepatocyte growth factor (HGF) | Matrix metalloproteinase-10 (MMP-10) |
| --- | --- | --- |
| Artemin (ARTN) | Interferon gamma (IFN-γ) | Monocyte chemotactic protein 1 (MCP-1) |
| Axin-1 (AXIN1) | Interleukin-1 alpha (IL-1α) | Monocyte chemotactic protein 2 (MCP-2) |
| Beta-nerve growth factor (β-NGF) | Interleukin-2 (IL-2) | Monocyte chemotactic protein 3 (MCP-3) |
| Caspase-8 (CASP-8) | Interleukin-2 receptor subunit beta (IL-2RB) | Monocyte chemotactic protein 4 (MCP-4) |
| C-C motif chemokine 3 (CCL3) | Interleukin-4 (IL-4) | Natural killer cell receptor 2B4 (CD244) |
| C-C motif chemokine 4 (CCL4) | Interleukin-5 (IL-5) | Neurotrophin-3 (NT-3) |
| C-C motif chemokine 19 (CCL19) | Interleukin-6 (IL-6) | Neurturin (NRTN) |
| C-C motif chemokine 20 (CCL20) | Interleukin-7 (IL-7) | Oncostatin-M (OSM) |
| C-C motif chemokine 23 (CCL23) | Interleukin-8 (IL-8) | Osteoprotegerin (OPG) |
| C-C motif chemokine 25 (CCL25) | Interleukin-10 (IL-10) | Protein S100-A12 (EN-RAGE) |
| C-C motif chemokine 28 (CCL28) | Interleukin-10 receptor subunit alpha (IL-10RA) | Signaling lymphocytic activation molecule (SLAMF1) |
| CD40L receptor (CD40) | Interleukin-10 receptor subunit beta (IL-10RB) | SIR2-like protein 2 (SIRT2) |
| CUB domain-containing protein 1 (CDCP1) | Interleukin-12 subunit beta (IL-12B) | STAM-binding protein (STAMBP) |
| C-X-C motif chemokine 1 (CXCL1) | Interleukin-13 (IL-13) | Stem cell factor (SCF) |
| C-X-C motif chemokine 5 (CXCL5) | Interleukin-15 receptor subunit alpha (IL-15RA) | Sulfotransferase 1A1 (ST1A1) |
| C-X-C motif chemokine 6 (CXCL6) | Interleukin-17A (IL-17A) | T cell surface glycoprotein CD6 isoform (CD6) |
| C-X-C motif chemokine 9 (CXCL9) | Interleukin-17C (IL-17C) | T-cell surface glycoprotein CD5 (CD5) |
| C-X-C motif chemokine 10 (CXCL10) | Interleukin-18 (IL-18) | T-cell surface glycoprotein CD8 alpha chain (CD8A) |
| C-X-C motif chemokine 11 (CXCL11) | Interleukin-18 receptor 1 (IL-18R1) | Thymic stromal lymphopoietin (TSLP) |
| Cystatin D (CST5) | Programmed cell death 1 ligand 1 (PD-L1) | TNF-beta (TNF-β) |
| Delta and Notch-like epidermal growth factor-related receptor (DNER) | Interleukin-20 (IL-20) | TNF-related activation-induced cytokine (TRANCE) |
| Eotaxin (CCL11) | Interleukin-20 receptor subunit alpha (IL-20RA) | TNF-related apoptosis-inducing ligand (TRAIL) |
| Eukaryotic translation initiation factor 4E-binding protein 1 (4E-BP1) | Interleukin-22 receptor subunit alpha-1 (IL-22RA1) | Transforming growth factor alpha (TGF-α) |
| Fibroblast growth factor 5 (FGF-5) | Interleukin-24 (IL-24) | Tumor necrosis factor (Ligand) superfamily, member 12 (TWEAK) |
| Fibroblast growth factor 19 (FGF-19) | Interleukin-33 (IL-33) | Tumor necrosis factor (TNF-α) |
| Fibroblast growth factor 21 (FGF-21) | Latency-associated peptide transforming growth factor beta-1 (LAP TGF-β-1) | Tumor necrosis factor ligand superfamily member 14 (TNFSF14) |
| Fibroblast growth factor 23 (FGF-23) | Leukemia inhibitory factor (LIF) | Tumor necrosis factor receptor superfamily member 9 (TNFRSF9) |
| Fms-related tyrosine kinase 3 ligand (Flt3L) | Leukemia inhibitory factor receptor (LIF-R) | Urokinase-type plasminogen activator (uPA) |
| Fractalkine (CX3CL1) | Macrophage colony-stimulating factor 1 (CSF-1) | Vascular endothelial growth factor A (VEGF-A) |
| Glial cell line-derived neurotrophic factor (GDNF) | Matrix metalloproteinase-1 (MMP-1) |  |

For consistency, abbreviations provided by OLINK® proteomics have been modified.

**S2. Inflammatory markers not included in main analysis**

|  | Fasting | | ΔHyperinsulinemia | | | ΔHypoglycemia | | | ΔHyperglycemia | | |
| --- | --- | --- | --- | --- | --- | --- | --- | --- | --- | --- | --- |
|  | Lean | Obese | Lean |  | Obese | Lean |  | Obese | Lean |  | Obese |
| Chemokines |  |  |  |  |  |  |  |  |  |  |  |
| MCP-3 | 1.89(1.05) | 2.37(0.90) | -0.03(0.71) |  | 0.10(0.81) | 0.06(0.39) |  | 0.07(0.91) | -0.20(1.44) | †† | -0.20(1.43) |
| Cytokines |  |  |  |  |  |  |  |  |  |  |  |
| IL-1α | -0.78(0.52) | -0.69(0.39) | 0.07(0.73) |  | -0.13(0.92) | -0.25(0.76) |  | -0.01(0.25) | -0.04(0.99) |  | -0.27(0.85) |
| IL-2 | 2.00(0.27) | 2.00(0.48) | 0.08(0.71) |  | 0.01(0.53) | -0.11(0.55) |  | -0.05(0.64) | -0.10(1.00) |  | -0.03(0.92) |
| IL-4 | 1.77(0.95) | 2.00(1.12) | -0.10(1.32) |  | -0.20(1.49) | -0.12(1.39) |  | -0.07(1.05) | -0.26(1.03) | † | -0.16(0.88) |
| IL-5 | 1.61(3.2) | 2.54(2.49)* | -0.05(0.45) |  | -0.13(0.69) | 0.12(0.63) |  | -0.10(0.40) | -0.08(0.58) | † | -0.08(0.56) |
| IL-13 | 1.41(1.51) | 1.48(6.11) | -0.04(0.89) |  | -0.03(0.78) | 0.07(0.76) |  | -0.03(0.56) | -0.03(0.50) |  | 0.00(1.07) |
| IL-17A | 3.18(0.65) | 3.27(1.23) | 0.11(0.91) |  | -0.07(0.96) | -0.12(0.93) |  | 0.05(0.76) | -0.15(1.01) |  | -0.11(0.98) |
| IL-20 | 1.87(0.18) | 1.94(0.33)* | -0.09(0.61) |  | -0.11(0.48) | -0.03(0.55) |  | -0.03(0.17) | -0.13(0.43) |  | -0.12(0.87) |
| IL-24 | 2.53(0.87) | 2.9(2.47) | -0.01(1.21) |  | 0.04(1.48) | -0.53(3.79) |  | -0.04(0.85) | 0.00(1.81) |  | -0.04(0.83) |
| IL-33 | 2.30(0.32) | 2.34(0.54) | -0.08(0.48) |  | -0.06(0.91) | -0.12(0.41) |  | 0.08(0.52) | -0.12(0.85) |  | -0.24(0.99) |
| LIF | 0.73(0.34) | 0.74(0.57) | -0.08(0.79) |  | -0.05(0.67) | 0.15(0.57) |  | 0.04(0.89) | -0.05(0.90) |  | -0.08(0.45) |
| TSLP | 1.93(0.57) | 2.11(1.15) | 0.14(1.57) |  | -0.3(0.88) | -0.07(1.33) |  | -0.03(0.99) | -0.3(1.43) |  | -0.39(0.89) |
| Growth factors | |  |  |  |  |  |  |  |  |  |  |
| ARTN | 1.69(2.1) | 2.02(5.34) | 0.00(0.65) |  | -0.19(0.72) | 0.05(0.57) | † | 0.14(0.55) | 0.00(0.63) |  | -0.02(0.21) |
| β-NGF | -0.17(0.17) | -0.1(0.41) | 0.02(0.23) |  | -0.06(0.22) | 0.00(0.22) |  | -0.01(0.20) | -0.04(0.23) |  | 0.15(1.81) |
| FGF-5 | 2.36(0.52) | 2.17(0.80) | 0.00(0.20) |  | -0.04(0.39) | -0.01(0.57) |  | -0.08(0.36) | -0.16(0.42) | †† | -0.18(0.53) |
| FGF-23 | 1.39(1.64) | 1.62(0.59) | 0.01(0.57) |  | -0.16(0.66) | -0.03(0.82) |  | 0.00(0.30) | -0.16(0.88) |  | 0.05(0.52) |
| GDNF | 2.35(1.86) | 2.31(0.72) | 0.14(0.73) |  | -0.26(0.89)* | -0.05(0.49) |  | 0.22(0.37)* | -0.06(1.03) |  | -0.03(0.43) |
| NRTN | 1.59(0.78) | 1.83(0.39)* | 0.04(0.39) |  | -0.10(0.87) | 0.05(0.37) |  | -0.07(0.61) | -0.07(0.84) |  | -0.13(0.85) |
| Receptors | |  |  |  |  |  |  |  |  |  |  |
| IL-2RB | 2.07(0.71) | 2.09(2.82) | -0.01(0.85) |  | -0.21(1.34) | 0.01(0.73) |  | 0.20(0.65) | -0.27(0.83) |  | -0.15(0.58) |
| IL-10RA | 2.01(0.57) | 2.51(1.73)** | 0.05(0.35) |  | -0.05(0.29) | -0.04(0.32) |  | 0.03(0.34) | -0.08(0.73) |  | -0.26(1.57) |
| IL-20RA | 2.06(1.17) | 2.07(0.27) | 0.12(0.90) |  | -0.14(0.34) | -0.12(0.8) |  | 0.21(0.49) | -0.18(0.71) |  | 0.04(0.9) |
| IL-22RA1 | 2.41(5.14) | 2.63(0.96) | 0.06(1.74) |  | -0.02(1.45) | -0.35(1.7) |  | -0.23(0.66) | -0.26(0.89) | †† | -0.01(0.84) |

Data presented as median (range). * *p*<0.05, ***p*<0.01, group comparisons by Mann-Whitney U tests. † *p*<0.05, †† *p*<0.01, one sample Wilcoxon signed-rank tests for all subjects.

**S3. Spearman correlations between inflammatory responses to hyperinsulinemia, metabolic parameters and achieved insulin levels**

|  | BMI | Body fat | WHR | HbA1c | f-Glucose | HOMA-IR | M-value | CRP | Insulin |
| --- | --- | --- | --- | --- | --- | --- | --- | --- | --- |
| **Chemokines** |  |  |  |  |  |  |  |  |  |
| CCL3 | -0.19 | -0.37 | -0.04 | 0.10 | 0.13 | 0.02 | -0.17 | -0.20 | 0.14 |
| CCL4 | 0.03 | -0.10 | 0.10 | 0.48 | 0.24 | 0.07 | -0.28 | 0.05 | 0.15 |
| CCL11 | -0.36 | **-0.53** | -0.16 | 0.19 | 0.29 | -0.04 | -0.10 | -0.17 | 0.05 |
| CCL19 | -0.37 | **-0.53** | -0.21 | 0.09 | 0.29 | -0.05 | 0.00 | -0.22 | 0.05 |
| CCL20 | -0.19 | -0.07 | -0.29 | -0.05 | 0.00 | -0.20 | 0.33 | -0.25 | -0.10 |
| CCL23 | -0.40 | **-0.51** | -0.30 | 0.16 | 0.21 | -0.16 | 0.07 | -0.33 | -0.05 |
| CCL25 | -0.24 | -0.42 | -0.14 | 0.10 | 0.40 | -0.02 | -0.07 | -0.24 | 0.03 |
| CCL28 | -0.33 | -0.48 | -0.29 | 0.05 | 0.00 | -0.14 | 0.06 | -0.29 | -0.06 |
| CXCL1 | 0.05 | 0.09 | -0.08 | 0.13 | -0.12 | -0.06 | 0.17 | 0.17 | -0.02 |
| CXCL5 | 0.12 | 0.11 | 0.08 | 0.30 | 0.03 | 0.09 | -0.02 | 0.28 | 0.10 |
| CXCL6 | 0.08 | -0.05 | 0.10 | 0.30 | -0.02 | 0.06 | -0.07 | 0.08 | 0.12 |
| CXCL9 | -0.26 | -0.41 | -0.07 | 0.03 | 0.21 | 0.05 | -0.08 | -0.22 | 0.23 |
| CXCL10 | -0.08 | -0.28 | 0.10 | 0.45 | 0.37 | 0.19 | -0.35 | -0.04 | 0.25 |
| CXCL11 | 0.03 | -0.10 | 0.05 | 0.44 | 0.06 | 0.03 | -0.07 | 0.10 | 0.18 |
| CX3CL1 | -0.07 | -0.30 | 0.16 | 0.30 | 0.40 | 0.37 | -0.39 | -0.02 | 0.40 |
| MCP-1 | -0.28 | -0.41 | -0.07 | 0.20 | 0.07 | -0.19 | 0.05 | -0.49 | -0.04 |
| MCP-2 | -0.01 | -0.14 | 0.04 | **0.55** | 0.26 | 0.12 | -0.24 | 0.13 | 0.20 |
| MCP-4 | 0.19 | 0.00 | 0.31 | 0.40 | 0.29 | 0.22 | -0.27 | 0.24 | 0.30 |
| **Cytokines** |  |  |  |  |  |  |  |  |  |
| EN-RAGE | -0.22 | -0.21 | -0.34 | -0.07 | -0.19 | -0.21 | 0.17 | -0.36 | -0.15 |
| Flt3L | -0.34 | -0.42 | -0.24 | 0.33 | 0.15 | -0.10 | -0.02 | -0.29 | 0.19 |
| IFN- γ | -0.07 | -0.19 | -0.01 | 0.19 | 0.35 | 0.20 | -0.28 | -0.09 | 0.07 |
| IL-6 | -0.02 | 0.09 | -0.11 | -0.13 | -0.19 | -0.17 | 0.16 | -0.31 | -0.28 |
| IL-7 | -0.03 | -0.14 | 0.01 | 0.37 | 0.02 | -0.06 | 0.04 | 0.06 | 0.06 |
|  | BMI | Body fat | WHR | HbA1c | f-Glucose | HOMA-IR | M-value | CRP | Insulin |
| **Cytokines** |  |  |  |  |  |  |  |  |  |
| IL-8 | 0.18 | 0.00 | 0.33 | 0.33 | 0.21 | 0.16 | -0.35 | 0.12 | 0.29 |
| IL-10 | -0.19 | -0.37 | -0.05 | 0.32 | 0.26 | 0.20 | -0.31 | -0.06 | 0.18 |
| IL-12B | -0.37 | **-0.57** | -0.17 | 0.11 | 0.17 | -0.03 | -0.06 | -0.23 | 0.22 |
| IL-17C^a^ | 0.43 | 0.28 | 0.25 | -0.01 | **0.52** | 0.42 | -0.46 | 0.43 | 0.01 |
| IL-18 | -0.24 | -0.46 | -0.04 | 0.25 | 0.20 | 0.12 | -0.25 | -0.11 | 0.23 |
| OSM | 0.11 | -0.07 | 0.19 | 0.40 | 0.09 | 0.17 | -0.26 | -0.05 | 0.23 |
| TNF-α | -0.20 | -0.36 | -0.03 | 0.21 | 0.38 | 0.25 | -0.32 | -0.03 | 0.34 |
| TNF-β | -0.23 | -0.32 | -0.16 | 0.22 | 0.24 | -0.07 | -0.05 | -0.28 | 0.14 |
| TNFRSF9 | -0.35 | -0.45 | -0.17 | 0.14 | 0.12 | -0.10 | 0.05 | -0.39 | 0.08 |
| TNFSF14 | 0.09 | -0.09 | 0.19 | 0.33 | -0.07 | 0.04 | -0.15 | -0.10 | 0.26 |
| TRANCE | 0.03 | -0.11 | 0.13 | 0.12 | 0.22 | 0.14 | -0.03 | -0.18 | 0.12 |
| TWEAK | -0.40 | **-0.57** | -0.19 | 0.05 | 0.27 | -0.08 | 0.00 | -0.27 | 0.12 |
| **Enzymes** |  |  |  |  |  |  |  |  |  |
| ADA | 0.08 | -0.10 | 0.20 | 0.30 | -0.05 | 0.12 | -0.17 | -0.01 | 0.31 |
| CST5 | -0.32 | -0.42 | -0.25 | 0.12 | 0.12 | -0.12 | -0.02 | -0.31 | 0.01 |
| MMP-1 | 0.21 | 0.21 | 0.06 | 0.34 | -0.09 | 0.02 | 0.03 | 0.15 | 0.02 |
| MMP-10 | -0.29 | -0.43 | -0.22 | 0.10 | 0.14 | -0.13 | 0.06 | -0.34 | -0.01 |
| SIRT2 | 0.12 | 0.00 | 0.22 | 0.33 | -0.09 | 0.15 | -0.22 | 0.04 | 0.22 |
| ST1A1 | -0.01 | 0.15 | -0.04 | 0.05 | -0.32 | -0.17 | 0.27 | 0.00 | -0.07 |
| uPA | -0.27 | -0.48 | -0.04 | -0.07 | 0.26 | 0.07 | -0.12 | -0.20 | 0.20 |
| **Growth factors** | |  |  |  |  |  |  |  |  |
| CSF-1 | -0.21 | -0.39 | 0.01 | 0.00 | 0.28 | 0.12 | -0.22 | -0.18 | 0.23 |
| DNER | -0.37 | **-0.53** | -0.15 | 0.10 | 0.28 | -0.04 | -0.03 | -0.22 | 0.17 |
| FGF-19 | -0.44 | -0.45 | -0.38 | 0.36 | -0.26 | -0.40 | 0.25 | **-0.57** | -0.16 |
| FGF-21 | -0.08 | -0.24 | -0.01 | 0.39 | 0.42 | 0.25 | -0.33 | -0.03 | 0.17 |
| HGF | -0.06 | -0.30 | 0.16 | 0.38 | 0.28 | 0.13 | -0.34 | -0.08 | 0.23 |
|  | BMI | Body fat | WHR | HbA1c | f-Glucose | HOMA-IR | M-value | CRP | Insulin |
| **Growth factors** | |  |  |  |  |  |  |  |  |
| LAP TGF-β-1 | -0.27 | -0.37 | -0.12 | 0.26 | -0.20 | -0.28 | 0.23 | -0.32 | 0.11 |
| NT-3 | -0.11 | -0.19 | -0.02 | 0.02 | 0.20 | 0.12 | -0.24 | -0.01 | 0.07 |
| SCF | -0.17 | -0.29 | -0.12 | 0.16 | 0.49 | 0.12 | -0.14 | -0.07 | 0.09 |
| TGF- α | 0.02 | -0.15 | 0.04 | 0.25 | 0.40 | 0.17 | -0.18 | 0.15 | 0.06 |
| VEGF-A | -0.23 | -0.40 | 0.02 | -0.09 | -0.03 | -0.21 | 0.09 | -0.28 | 0.12 |
| **Intracellular signalling molecules** | | |  |  |  |  |  |  |  |
| 4E-BP1 | 0.22 | 0.08 | 0.30 | 0.33 | -0.03 | 0.22 | -0.27 | 0.07 | 0.28 |
| AXIN1 | -0.09 | -0.06 | -0.15 | 0.21 | -0.30 | -0.19 | 0.22 | -0.05 | 0.00 |
| CASP-8 | 0.12 | 0.04 | 0.11 | 0.32 | -0.20 | 0.17 | -0.22 | -0.01 | 0.27 |
| STAMBP | 0.09 | -0.02 | 0.17 | 0.24 | -0.23 | 0.07 | -0.15 | -0.05 | 0.19 |
| **Receptors** |  |  |  |  |  |  |  |  |  |
| CD5 | -0.05 | -0.22 | 0.02 | 0.31 | 0.29 | 0.19 | -0.34 | -0.07 | 0.01 |
| CD6 | -0.22 | -0.32 | -0.12 | 0.23 | 0.10 | 0.05 | -0.17 | -0.21 | 0.13 |
| CD8A | -0.25 | -0.39 | -0.08 | 0.00 | 0.25 | 0.00 | -0.09 | -0.23 | 0.15 |
| CD40 | **-0.52** | **-0.60** | -0.43 | 0.15 | -0.36 | -0.41 | 0.32 | **-0.59** | -0.06 |
| CD244 | -0.43 | -0.48 | -0.45 | 0.22 | -0.23 | -0.30 | 0.27 | **-0.51** | -0.01 |
| CDCP1 | -0.28 | -0.18 | -0.41 | 0.45 | 0.13 | -0.27 | 0.11 | -0.22 | -0.08 |
| IL-10RB | -0.26 | -0.48 | -0.10 | 0.38 | 0.28 | 0.14 | -0.25 | -0.03 | 0.30 |
| IL-15RA | -0.19 | -0.38 | -0.16 | **0.51** | 0.28 | 0.12 | -0.26 | -0.08 | 0.12 |
| IL-18R1 | -0.30 | **-0.50** | -0.10 | 0.14 | 0.36 | 0.05 | -0.16 | -0.14 | 0.20 |
| LIF-R | -0.14 | -0.34 | 0.11 | 0.03 | 0.40 | 0.19 | -0.24 | -0.17 | 0.22 |
| OPG | -0.26 | -0.44 | -0.08 | 0.06 | 0.41 | 0.10 | -0.12 | -0.09 | 0.24 |
| PD-L1 | **-0.50** | **-0.72** | -0.25 | 0.04 | -0.10 | -0.21 | 0.13 | -0.48 | 0.09 |
| SLAMF1 | -0.17 | -0.20 | -0.23 | 0.14 | 0.02 | 0.00 | 0.17 | -0.20 | 0.17 |
| TRAIL | -0.15 | -0.34 | 0.04 | 0.11 | 0.48 | 0.22 | -0.29 | -0.09 | 0.22 |

Inflammatory responses are $\Delta$ with respect to fasting levels. Insulin is levels at the end of the hyperinsulinemic-euglucemic phase (T2). Body fat= in % as obtained by bioimpedance, WHR= Waist-hip ratio, f-Glucose= fasting plasma glucose. All metabolic parameters were obtained at first occasion. Background in three color scale where blue refer to negative coefficients and red refer to positive coefficients, with increasing intensity with higher magnitude. White refers to a coefficient of 0. Significant coefficients are typed in **bold**. ^a^Data missing for one subject.

**S4. Spearman Correlations between inflammatory responses to hypoglycemia, metabolic parameters and hormonal responses**

|  | BMI | Body fat | WHR | HbA1c | f-Glucose | HOMA | M-value | CRP | Glucagon | Cortisol | ACTH | GH | Insulin |
| --- | --- | --- | --- | --- | --- | --- | --- | --- | --- | --- | --- | --- | --- |
| **Chemokines** |  |  |  |  |  |  |  |  |  |  |  |  |  |
| CCL3 | -0.24 | -0.11 | -0.32 | 0.16 | -0.47 | -0.11 | 0.02 | -0.10 | 0.18 | -0.33 | -0.28 | 0.11 | -0.11 |
| CCL4 | -0.23 | -0.19 | -0.19 | 0.00 | -0.49 | -0.02 | -0.02 | -0.10 | 0.39 | -0.27 | -0.24 | 0.11 | 0.04 |
| CCL11 | -0.08 | -0.01 | -0.13 | -0.04 | **-0.55** | -0.09 | 0.02 | -0.17 | 0.40 | -0.04 | -0.12 | 0.39 | -0.12 |
| CCL19 | -0.05 | 0.06 | -0.09 | 0.00 | **-0.52** | -0.06 | 0.05 | -0.08 | 0.27 | -0.05 | -0.11 | 0.22 | -0.17 |
| CCL20 | -0.05 | 0.01 | -0.14 | 0.20 | -0.31 | 0.01 | -0.11 | 0.02 | 0.17 | -0.05 | -0.02 | 0.38 | -0.20 |
| CCL23 | 0.01 | 0.13 | -0.11 | 0.05 | **-0.56** | -0.08 | 0.07 | -0.13 | 0.29 | -0.01 | -0.06 | 0.17 | -0.13 |
| CCL25 | -0.10 | 0.07 | -0.26 | 0.08 | **-0.65** | -0.20 | 0.13 | -0.28 | 0.27 | -0.07 | -0.17 | 0.15 | -0.25 |
| CCL28 | -0.25 | -0.06 | -0.37 | -0.08 | **-0.76** | -0.30 | 0.32 | -0.26 | 0.21 | -0.31 | -0.23 | 0.09 | -0.25 |
| CXCL1 | -0.10 | -0.27 | 0.12 | **-0.55** | -0.19 | 0.06 | -0.09 | -0.21 | **0.54** | 0.09 | -0.05 | 0.34 | 0.25 |
| CXCL5 | -0.27 | -0.44 | 0.04 | -0.49 | -0.25 | -0.02 | -0.07 | -0.30 | **0.58** | -0.04 | -0.18 | 0.32 | 0.24 |
| CXCL6 | -0.03 | -0.08 | 0.06 | -0.24 | -0.15 | 0.13 | -0.30 | -0.04 | 0.36 | 0.10 | -0.09 | 0.37 | 0.12 |
| CXCL9 | 0.11 | 0.18 | -0.03 | 0.07 | -0.42 | 0.05 | -0.03 | -0.03 | 0.25 | 0.15 | 0.07 | 0.23 | -0.20 |
| CXCL10 | 0.14 | 0.26 | -0.04 | -0.02 | -0.49 | -0.02 | -0.02 | -0.01 | 0.15 | -0.07 | -0.14 | -0.07 | 0.03 |
| CXCL11 | 0.09 | 0.05 | 0.03 | -0.28 | -0.07 | 0.16 | -0.24 | 0.08 | 0.36 | 0.11 | 0.14 | 0.20 | 0.14 |
| CX3CL1 | -0.21 | 0.04 | -0.45 | 0.07 | **-0.70** | -0.38 | 0.29 | -0.30 | 0.24 | -0.21 | -0.25 | 0.07 | -0.36 |
| MCP-1 | -0.05 | 0.05 | -0.22 | -0.04 | **-0.50** | -0.10 | 0.18 | -0.10 | 0.15 | 0.01 | 0.02 | 0.23 | -0.33 |
| MCP-2 | -0.07 | -0.03 | -0.16 | -0.24 | **-0.53** | -0.08 | 0.07 | -0.19 | 0.42 | -0.07 | -0.20 | 0.25 | -0.04 |
| MCP-4 | -0.25 | -0.17 | -0.32 | -0.37 | **-0.59** | -0.24 | 0.20 | -0.28 | 0.39 | -0.22 | -0.39 | 0.03 | -0.15 |
|  | BMI | Body fat | WHR | HbA1c | f-Glucose | HOMA | M-value | CRP | Glucagon | Cortisol | ACTH | GH | Insulin |
| **Cytokines** |  |  |  |  |  |  |  |  |  |  |  |  |  |
| EN-RAGE | 0.21 | 0.22 | 0.05 | -0.03 | 0.11 | 0.31 | -0.11 | 0.27 | -0.23 | 0.12 | 0.07 | 0.05 | 0.23 |
| Flt3L | -0.27 | -0.10 | -0.36 | -0.15 | **-0.80** | -0.37 | 0.34 | -0.39 | 0.33 | -0.26 | -0.32 | 0.23 | -0.27 |
| IFN- γ | -0.17 | -0.02 | -0.32 | -0.09 | **-0.61** | -0.23 | 0.18 | -0.29 | 0.27 | -0.05 | -0.20 | 0.25 | -0.26 |
| IL-6 | 0.14 | 0.09 | 0.03 | -0.36 | -0.10 | 0.04 | 0.17 | 0.00 | 0.44 | 0.12 | 0.28 | 0.05 | 0.05 |
| IL-7 | -0.14 | -0.27 | 0.01 | -0.44 | -0.09 | 0.04 | -0.14 | -0.16 | 0.34 | -0.05 | -0.10 | 0.33 | 0.23 |
| IL-8 | -0.20 | -0.15 | -0.19 | -0.12 | -0.42 | -0.04 | 0.00 | -0.11 | 0.19 | -0.12 | -0.27 | 0.06 | -0.19 |
| IL-10 | 0.00 | -0.05 | 0.11 | -0.18 | -0.33 | -0.07 | 0.17 | -0.32 | 0.44 | 0.48 | 0.14 | 0.49 | -0.32 |
| IL-12B | -0.24 | -0.10 | -0.27 | 0.11 | **-0.63** | -0.25 | 0.16 | -0.32 | 0.33 | -0.12 | -0.26 | 0.28 | -0.25 |
| IL-17C | -0.38 | -0.17 | -0.43 | -0.20 | **-0.77** | -0.41 | 0.50 | -0.41 | 0.14 | -0.26 | -0.42 | 0.06 | -0.29 |
| IL-18 | -0.14 | -0.01 | -0.17 | -0.08 | **-0.61** | -0.13 | 0.07 | -0.21 | 0.40 | -0.09 | -0.18 | 0.34 | -0.14 |
| OSM | **-0.56** | **-0.53** | -0.37 | -0.32 | -0.46 | -0.47 | 0.32 | -0.37 | 0.47 | -0.42 | **-0.52** | -0.20 | -0.12 |
| TNF-α | -0.06 | 0.04 | -0.22 | 0.00 | **-0.56** | -0.06 | 0.09 | -0.08 | 0.32 | -0.14 | -0.13 | -0.02 | -0.11 |
| TNF-β | -0.27 | -0.07 | -0.39 | -0.09 | **-0.76** | -0.42 | 0.39 | -0.45 | 0.37 | -0.15 | -0.29 | 0.17 | -0.36 |
| TNFRSF9 | -0.12 | 0.04 | -0.32 | -0.05 | **-0.65** | -0.23 | 0.16 | -0.24 | 0.14 | -0.20 | -0.22 | 0.16 | -0.27 |
| TNFSF14 | **-0.52** | **-0.51** | -0.34 | -0.47 | **-0.62** | -0.29 | 0.30 | -0.33 | 0.29 | -0.39 | **-0.51** | 0.05 | -0.14 |
| TRANCE | -0.15 | 0.01 | -0.24 | 0.11 | -0.42 | -0.22 | -0.02 | -0.27 | 0.26 | 0.05 | -0.24 | 0.47 | -0.39 |
| TWEAK | -0.28 | -0.12 | -0.40 | -0.02 | **-0.71** | -0.28 | 0.23 | -0.24 | 0.24 | -0.27 | -0.33 | 0.12 | -0.26 |
| **Enzymes** |  |  |  |  |  |  |  |  |  |  |  |  |  |
| ADA | -0.19 | -0.19 | -0.17 | -0.26 | -0.30 | -0.17 | 0.19 | -0.36 | 0.45 | -0.09 | -0.32 | -0.04 | -0.09 |
| CST5 | -0.10 | 0.04 | -0.28 | -0.05 | **-0.59** | -0.15 | 0.15 | -0.15 | 0.20 | -0.16 | -0.17 | 0.07 | -0.19 |
| MMP-1 | -0.12 | -0.28 | 0.05 | **-0.58** | -0.15 | -0.04 | -0.07 | -0.11 | 0.47 | -0.10 | -0.13 | 0.21 | 0.22 |
| MMP-10 | -0.29 | -0.12 | -0.42 | -0.09 | **-0.75** | -0.32 | 0.28 | -0.28 | 0.28 | -0.33 | -0.34 | 0.14 | -0.24 |
| SIRT2 | -0.29 | **-0.51** | -0.01 | -0.46 | 0.03 | -0.07 | 0.09 | -0.33 | 0.42 | 0.01 | -0.12 | 0.20 | 0.13 |
| ST1A1 | -0.17 | -0.43 | 0.16 | -0.19 | 0.17 | 0.19 | -0.27 | -0.10 | 0.16 | -0.04 | -0.06 | 0.24 | 0.38 |
| uPA | -0.23 | -0.10 | -0.24 | -0.06 | **-0.55** | -0.18 | 0.21 | -0.18 | 0.27 | -0.04 | -0.22 | 0.25 | -0.27 |
|  |  |  |  |  |  |  |  |  |  |  |  |  |  |
|  | BMI | Body fat | WHR | HbA1c | f-Glucose | HOMA | M-value | CRP | Glucagon | Cortisol | ACTH | GH | Insulin |
| **Growth factors** | |  |  |  |  |  |  |  |  |  |  |  |  |
| CSF-1 | -0.30 | -0.12 | -0.39 | -0.07 | **-0.75** | -0.38 | 0.34 | -0.38 | 0.32 | -0.18 | -0.30 | 0.22 | -0.37 |
| DNER | -0.24 | -0.07 | -0.35 | -0.10 | **-0.73** | -0.32 | 0.26 | -0.35 | 0.33 | -0.18 | -0.25 | 0.31 | -0.28 |
| FGF-19 | -0.03 | 0.03 | -0.20 | **0.58** | -0.27 | -0.17 | -0.02 | -0.37 | 0.04 | -0.12 | -0.21 | 0.08 | -0.29 |
| FGF-21 | **-0.53** | -0.32 | **-0.65** | 0.19 | **-0.65** | **-0.52** | 0.41 | -0.44 | -0.05 | -0.49 | -0.42 | -0.15 | **-0.55** |
| HGF | -0.47 | -0.35 | **-0.51** | -0.12 | **-0.77** | -0.39 | 0.32 | -0.40 | 0.29 | -0.49 | **-0.56** | 0.01 | -0.18 |
| LAP TGF-β-1 | -0.03 | -0.04 | -0.05 | -0.21 | -0.32 | 0.04 | -0.10 | -0.05 | 0.29 | 0.05 | -0.11 | 0.31 | -0.04 |
| NT-3 | -0.10 | 0.00 | -0.19 | -0.17 | -0.43 | -0.08 | 0.19 | -0.05 | 0.12 | -0.05 | -0.07 | 0.19 | -0.25 |
| SCF | -0.32 | -0.14 | -0.37 | -0.04 | **-0.78** | -0.37 | 0.27 | -0.39 | 0.40 | -0.28 | -0.36 | 0.29 | -0.24 |
| TGF- α | -0.03 | 0.11 | -0.19 | 0.25 | -0.4 | 0.02 | -0.17 | 0.03 | -0.07 | -0.18 | -0.19 | 0.15 | -0.14 |
| VEGF-A | -0.19 | -0.11 | -0.24 | -0.13 | **-0.64** | -0.16 | 0.16 | -0.19 | 0.30 | -0.19 | -0.32 | 0.11 | -0.15 |
| **Intracellular signalling molecules** | | |  |  |  |  |  |  |  |  |  |  |  |
| 4E-BP1 | **-0.51** | **-0.54** | -0.36 | -0.28 | -0.27 | -0.41 | 0.32 | -0.44 | 0.47 | -0.37 | -0.45 | -0.02 | -0.08 |
| AXIN1 | -0.29 | -0.49 | 0.17 | **-0.57** | 0.02 | 0.04 | 0.00 | -0.24 | 0.42 | 0.07 | -0.08 | 0.23 | 0.36 |
| CASP-8 | -0.47 | -0.49 | -0.39 | -0.32 | -0.33 | -0.36 | 0.32 | -0.40 | 0.44 | -0.42 | -0.50 | -0.05 | -0.10 |
| STAMBP | -0.30 | **-0.52** | 0.00 | -0.37 | 0.03 | -0.07 | 0.03 | -0.32 | 0.37 | 0.03 | -0.18 | 0.25 | 0.02 |
| **Receptors** |  |  |  |  |  |  |  |  |  |  |  |  |  |
| CD5 | -0.26 | -0.09 | -0.31 | 0.01 | **-0.64** | -0.26 | 0.17 | -0.31 | 0.34 | -0.07 | -0.26 | 0.32 | -0.29 |
| CD6 | -0.04 | 0.07 | -0.17 | -0.04 | -0.42 | -0.08 | 0.00 | -0.07 | 0.27 | 0.10 | 0.00 | 0.45 | -0.32 |
| CD8A | -0.18 | -0.04 | -0.27 | -0.08 | **-0.70** | -0.23 | 0.24 | -0.26 | 0.31 | -0.14 | -0.21 | 0.18 | -0.27 |
| CD40 | 0.14 | 0.15 | 0.15 | -0.05 | -0.21 | 0.26 | -0.20 | 0.07 | -0.03 | 0.12 | 0.00 | 0.24 | -0.03 |
| CD244 | -0.10 | -0.02 | -0.07 | -0.05 | -0.4 | 0.07 | -0.09 | 0.02 | 0.24 | -0.10 | -0.21 | 0.22 | 0.00 |
| CDCP1 | 0.18 | 0.21 | -0.01 | 0.08 | -0.16 | 0.25 | -0.32 | 0.15 | 0.07 | -0.01 | 0.04 | 0.22 | 0.08 |
| IL-10RB | -0.21 | -0.07 | -0.27 | 0.04 | -0.47 | -0.2 | 0.12 | -0.25 | 0.34 | 0.07 | -0.16 | 0.41 | -0.28 |
| IL-15RA | -0.24 | -0.16 | -0.27 | 0.20 | -0.5 | -0.25 | 0.18 | -0.43 | **0.52** | -0.23 | -0.32 | 0.01 | 0.09 |
| IL-18R1 | -0.24 | -0.08 | -0.29 | -0.01 | **-0.68** | -0.27 | 0.18 | -0.34 | 0.34 | -0.11 | -0.25 | 0.37 | -0.28 |
| LIF-R | -0.26 | -0.08 | -0.35 | 0.07 | **-0.65** | -0.33 | 0.23 | -0.33 | 0.29 | -0.11 | -0.25 | 0.31 | -0.34 |
|  | BMI | Body fat | WHR | HbA1c | f-Glucose | HOMA | M-value | CRP | Glucagon | Cortisol | ACTH | GH | Insulin |
| **Receptors** |  |  |  |  |  |  |  |  |  |  |  |  |  |
| OPG | -0.32 | -0.16 | -0.38 | -0.11 | **-0.75** | -0.34 | 0.35 | -0.32 | 0.32 | -0.26 | -0.33 | 0.14 | -0.28 |
| PD-L1 | -0.27 | -0.13 | -0.21 | 0.17 | -0.4 | -0.1 | -0.04 | -0.21 | 0.15 | -0.14 | -0.36 | 0.25 | -0.09 |
| SLAMF1 | -0.29 | -0.13 | **-0.52** | 0.43 | -0.12 | -0.24 | 0.27 | -0.02 | -0.16 | -0.38 | -0.13 | -0.15 | -0.20 |
| TRAIL | -0.11 | 0.04 | -0.16 | -0.08 | **-0.63** | -0.19 | 0.09 | -0.28 | 0.45 | 0.01 | -0.19 | 0.34 | -0.24 |

Inflammatory responses are $\Delta$ with respect to levels at hyperinsulinemia. Hormonal responses are $\Delta$AUC or AUC (insulin) of hypoglycemic phase (from hyperinsulinemia to hypoglycemia (80-185 min of hypoglycemic clamp). Body fat= in % as obtained by bioimpedance, WHR= Waist-hip ratio, f-Glucose= fasting plasma glucose, HOMA= HOMA-IR, GH= growth hormone. All metabolic parameters were obtained at first occasion. Background in three color scale where blue refers to negative coefficients and red refers to positive coefficients, increasing intensity with higher magnitude. White refers to a coefficient of 0. Significant coefficients are typed in **bold**. ^a^Data missing for one subject.

**S5. Spearman correlations between inflammatory responses to hyperglycemia, metabolic parameters and hormonal responses**

|  | | BMI | | | Body fat | | | WHR | | | HbA1c | | | f-Glucose | | | | HOMA | | | | M-value | | | | CRP | | | | Glucagon | | | | Cortisol | | | | ACTH | | | | GH | | | Insulin | | |  |
| --- | --- | --- | --- | --- | --- | --- | --- | --- | --- | --- | --- | --- | --- | --- | --- | --- | --- | --- | --- | --- | --- | --- | --- | --- | --- | --- | --- | --- | --- | --- | --- | --- | --- | --- | --- | --- | --- | --- | --- | --- | --- | --- | --- | --- | --- | --- | --- | --- |
| **Chemokines** | |  | |  | | |  | | |  | |  | | | |  | | | |  | | | |  | | |  | | | |  | | | |  | | | |  | | | |  | | | |  | |
| CCL3 | | -0.38 | | | -0.23 | | | -0.39 | | | 0.15 | | | 0.04 | | | | -0.37 | | | | 0.14 | | | | -0.34 | | | | 0.35 | | | | 0.27 | | | | 0.33 | | | | 0.01 | | | -0.38 | | |  |
| CCL4 | | -0.11 | | | -0.01 | | | -0.23 | | | 0.30 | | | 0.28 | | | | -0.19 | | | | 0.09 | | | | -0.20 | | | | 0.43 | | | | 0.31 | | | | 0.38 | | | | 0.09 | | | -0.28 | | |  |
| CCL11 | | -0.25 | | | -0.10 | | | -0.43 | | | 0.33 | | | -0.02 | | | | -0.41 | | | | 0.16 | | | | -0.46 | | | | 0.22 | | | | 0.41 | | | | 0.36 | | | | 0.12 | | | -0.46 | | |  |
| CCL19 | | -0.22 | | | -0.04 | | | -0.35 | | | 0.17 | | | 0.05 | | | | -0.31 | | | | 0.15 | | | | -0.28 | | | | 0.41 | | | | 0.22 | | | | **0.52** | | | | 0.10 | | | -0.24 | | |  |
| CCL20 | | -0.07 | | | -0.06 | | | -0.15 | | | 0.02 | | | -0.01 | | | | -0.19 | | | | 0.17 | | | | -0.21 | | | | 0.35 | | | | **0.62** | | | | 0.34 | | | | 0.16 | | | -0.46 | | |  |
| CCL23 | | **-0.53** | | | -0.42 | | | -0.46 | | | -0.12 | | | -0.05 | | | | -0.49 | | | | 0.30 | | | | **-0.53** | | | | 0.03 | | | | 0.15 | | | | 0.16 | | | | 0.01 | | | **-0.51** | | |  |
| CCL25 | | 0.18 | | | 0.23 | | | 0.01 | | | 0.16 | | | 0.37 | | | | 0.00 | | | | -0.13 | | | | 0.09 | | | | **0.55** | | | | 0.36 | | | | 0.47 | | | | 0.28 | | | -0.17 | | |  |
| CCL28 | | -0.24 | | | -0.37 | | | -0.05 | | | -0.12 | | | 0.03 | | | | -0.36 | | | | 0.22 | | | | -0.24 | | | | -0.10 | | | | 0.09 | | | | 0.08 | | | | 0.10 | | | **-0.51** | | |  |
| CXCL1 | | -0.04 | | | -0.02 | | | 0.17 | | | -0.48 | | | 0.12 | | | | 0.02 | | | | 0.02 | | | | -0.24 | | | | -0.13 | | | | 0.14 | | | | -0.08 | | | | -0.20 | | | -0.14 | | |  |
| CXCL5 | | 0.13 | | | 0.07 | | | 0.42 | | | -0.17 | | | 0.29 | | | | 0.16 | | | | -0.21 | | | | -0.08 | | | | 0.01 | | | | 0.12 | | | | 0.04 | | | | -0.12 | | | -0.02 | | |  |
| CXCL6 | | -0.15 | | | -0.05 | | | -0.02 | | | -0.29 | | | 0.16 | | | | -0.19 | | | | 0.15 | | | | -0.17 | | | | 0.03 | | | | 0.12 | | | | 0.14 | | | | 0.02 | | | -0.28 | | |  |
| CXCL9 | | -0.27 | | | -0.11 | | | -0.35 | | | 0.10 | | | 0.08 | | | | -0.39 | | | | 0.19 | | | | -0.40 | | | | 0.22 | | | | 0.29 | | | | 0.36 | | | | 0.22 | | | -0.46 | | |  |
| CXCL10 | | 0.08 | | | 0.15 | | | 0.03 | | | 0.32 | | | 0.22 | | | | -0.18 | | | | -0.15 | | | | -0.08 | | | | 0.35 | | | | 0.32 | | | | **0.51** | | | | 0.27 | | | -0.32 | | |  |
| CXCL11 | | -0.35 | | | -0.35 | | | -0.13 | | | -0.15 | | | 0.12 | | | | -0.27 | | | | 0.20 | | | | -0.39 | | | | -0.03 | | | | -0.06 | | | | -0.05 | | | | -0.11 | | | -0.27 | | |  |
| CX3CL1 | | 0.15 | | | 0.25 | | | 0.22 | | | -0.07 | | | -0.02 | | | | -0.06 | | | | 0.05 | | | | 0.01 | | | | 0.24 | | | | 0.09 | | | | 0.33 | | | | 0.10 | | | -0.05 | | |  |
| MCP-1 | | -0.16 | | | 0.04 | | | -0.34 | | | 0.07 | | | -0.16 | | | | -0.32 | | | | 0.19 | | | | -0.36 | | | | 0.19 | | | | 0.46 | | | | 0.49 | | | | 0.04 | | | -0.21 | | |  |
| MCP-2 | | -0.30 | | | -0.14 | | | -0.29 | | | 0.17 | | | 0.04 | | | | -0.45 | | | | 0.30 | | | | -0.37 | | | | 0.12 | | | | 0.21 | | | | 0.45 | | | | -0.05 | | | -0.44 | | |  |
| MCP-4 | | -0.43 | | | -0.36 | | | -0.28 | | | -0.11 | | | 0.01 | | | | -0.43 | | | | 0.37 | | | | -0.48 | | | | 0.00 | | | | 0.27 | | | | 0.14 | | | | -0.08 | | | **-0.52** | | |  |
| **Cytokines** | |  | |  | | |  | | |  | |  | | | |  | | | |  | | | |  | | |  | | | |  | | | |  | | | |  | | | |  | | | |  | |
| EN-RAGE | | 0.04 | | | -0.06 | | | 0.13 | | | 0.21 | | | 0.09 | | | | 0.04 | | | | -0.31 | | | | -0.13 | | | | 0.10 | | | | -0.02 | | | | 0.07 | | | | -0.19 | | | 0.09 | | |  |
| Flt3L | | -0.21 | | | 0.06 | | | -0.30 | | | -0.05 | | | -0.35 | | | | **-0.55** | | | | 0.37 | | | | -0.43 | | | | 0.07 | | | | 0.43 | | | | **0.64** | | | | 0.21 | | | **-0.52** | | |  |
| IFN- γ | | -0.14 | | | 0.01 | | | -0.46 | | | -0.03 | | | -0.24 | | | | -0.44 | | | | 0.24 | | | | -0.30 | | | | 0.07 | | | | **0.74** | | | | 0.36 | | | | 0.35 | | | **-0.57** | | |  |
| IL-6 | | -0.05 | | | 0.16 | | | -0.26 | | | -0.20 | | | -0.27 | | | | -0.46 | | | | 0.34 | | | | -0.21 | | | | -0.17 | | | | 0.49 | | | | **0.56** | | | | 0.43 | | | -0.40 | | |  |
| IL-7 | | -0.04 | | | -0.11 | | | 0.10 | | | -0.20 | | | 0.34 | | | | -0.05 | | | | 0.16 | | | | 0.04 | | | | 0.13 | | | | 0.04 | | | | 0.12 | | | | 0.20 | | | -0.18 | | |  |
|  | | BMI | | | Body fat | | | WHR | | | HbA1c | | | f-Glucose | | | | HOMA | | | | M-value | | | | CRP | | | | Glucagon | | | | Cortisol | | | | ACTH | | | | GH | | | Insulin | | |  |
| **Cytokines** | |  | |  | | |  | | |  | |  | | | |  | | | |  | | | |  | | |  | | | |  | | | |  | | | |  | | | |  | | | |  | |
| IL-8 | | 0.08 | | | 0.03 | | | 0.07 | | | 0.04 | | | 0.13 | | | | -0.11 | | | | -0.06 | | | | -0.09 | | | | 0.19 | | | | **0.52** | | | | 0.19 | | | | 0.23 | | | -0.37 | | |  |
| IL-10 | | -0.26 | | | -0.29 | | | -0.10 | | | -0.14 | | | -0.11 | | | | -0.32 | | | | 0.09 | | | | **-0.53** | | | | -0.09 | | | | 0.49 | | | | 0.13 | | | | 0.22 | | | **-0.53** | | |  |
| IL-12B | | -0.36 | | | -0.14 | | | **-0.50** | | | -0.18 | | | -0.19 | | | | **-0.52** | | | | **0.51** | | | | **-0.56** | | | | 0.06 | | | | 0.49 | | | | 0.44 | | | | 0.18 | | | **-0.55** | | |  |
| IL-17C | | 0.19 | | | 0.19 | | | 0.40 | | | -0.35 | | | 0.22 | | | | 0.08 | | | | -0.19 | | | | 0.05 | | | | 0.08 | | | | 0.01 | | | | 0.27 | | | | -0.02 | | | -0.04 | | |  |
| IL-18 | | -0.29 | | | -0.22 | | | -0.15 | | | 0.00 | | | 0.08 | | | | -0.23 | | | | 0.06 | | | | -0.43 | | | | 0.16 | | | | 0.23 | | | | 0.14 | | | | -0.06 | | | -0.27 | | |  |
| OSM | | -0.14 | | | -0.17 | | | 0.13 | | | -0.30 | | | -0.10 | | | | -0.20 | | | | 0.18 | | | | -0.26 | | | | -0.29 | | | | 0.19 | | | | -0.04 | | | | 0.08 | | | -0.33 | | |  |
| TNF-α | | -0.19 | | | -0.18 | | | -0.12 | | | 0.19 | | | -0.08 | | | | -0.41 | | | | 0.22 | | | | -0.44 | | | | -0.01 | | | | 0.25 | | | | 0.42 | | | | 0.21 | | | -0.42 | | |  |
| TNF-β | | 0.02 | | | -0.01 | | | 0.14 | | | 0.20 | | | 0.08 | | | | -0.03 | | | | 0.01 | | | | -0.11 | | | | 0.34 | | | | 0.21 | | | | 0.14 | | | | 0.12 | | | -0.20 | | |  |
| TNFRSF9 | | -0.10 | | | -0.18 | | | -0.12 | | | 0.26 | | | 0.12 | | | | -0.17 | | | | 0.04 | | | | -0.30 | | | | 0.30 | | | | 0.37 | | | | 0.33 | | | | 0.26 | | | -0.32 | | |  |
| TNFSF14 | | -0.02 | | | 0.00 | | | 0.07 | | | 0.23 | | | 0.23 | | | | -0.20 | | | | 0.03 | | | | -0.18 | | | | 0.17 | | | | 0.20 | | | | 0.41 | | | | 0.28 | | | -0.34 | | |  |
| TRANCE | | -0.22 | | | -0.07 | | | -0.31 | | | 0.26 | | | -0.33 | | | | -0.46 | | | | 0.27 | | | | **-0.54** | | | | 0.10 | | | | **0.58** | | | | **0.59** | | | | 0.07 | | | -0.46 | | |  |
| TWEAK | | -0.18 | | | -0.08 | | | -0.30 | | | -0.06 | | | 0.12 | | | | -0.34 | | | | 0.32 | | | | -0.30 | | | | 0.20 | | | | 0.15 | | | | 0.41 | | | | 0.32 | | | -0.33 | | |  |
| **Enzymes** | |  | |  | | |  | | |  | |  | | | |  | | | |  | | | |  | | |  | | | |  | | | |  | | | |  | | | |  | | | |  | |
| ADA | | -0.27 | | | -0.41 | | | 0.12 | | | -0.07 | | | -0.02 | | | | -0.23 | | | | 0.04 | | | | -0.50 | | | | -0.15 | | | | 0.22 | | | | -0.06 | | | | -0.10 | | | -0.38 | | |  |
| CST5 | | -0.20 | | | -0.06 | | | -0.23 | | | 0.11 | | | 0.07 | | | | -0.24 | | | | 0.03 | | | | -0.31 | | | | 0.32 | | | | 0.21 | | | | 0.39 | | | | 0.11 | | | -0.27 | | |  |
| MMP-1 | | -0.19 | | | -0.16 | | | -0.14 | | | 0.09 | | | 0.06 | | | | -0.25 | | | | 0.22 | | | | -0.34 | | | | 0.01 | | | | 0.23 | | | | 0.05 | | | | -0.09 | | | -0.36 | | |  |
| MMP-10 | | -0.19 | | | -0.09 | | | -0.35 | | | 0.04 | | | 0.02 | | | | -0.36 | | | | 0.28 | | | | -0.33 | | | | 0.20 | | | | 0.19 | | | | 0.47 | | | | 0.18 | | | -0.32 | | |  |
| SIRT2 | | -0.05 | | | -0.12 | | | 0.23 | | | -0.08 | | | 0.14 | | | | 0.08 | | | | -0.11 | | | | -0.16 | | | | 0.05 | | | | 0.20 | | | | -0.11 | | | | -0.23 | | | -0.07 | | |  |
| ST1A1 | | 0.12 | | | 0.20 | | | 0.07 | | | 0.17 | | | 0.38 | | | | 0.12 | | | | -0.14 | | | | -0.04 | | | | 0.37 | | | | 0.28 | | | | 0.25 | | | | -0.04 | | | -0.08 | | |  |
| uPA | | 0.06 | | | 0.16 | | | -0.08 | | | 0.18 | | | 0.11 | | | | -0.04 | | | | -0.17 | | | | -0.16 | | | | 0.48 | | | | 0.42 | | | | 0.49 | | | | 0.15 | | | -0.09 | | |  |
| **Growth factors** |  | |  | | |  | | |  | | | |  | | | |  | | | |  | | | |  | | | |  | | | |  | | | |  | | | |  | | | | |  | | |
| CSF-1 | | -0.11 | | | -0.05 | | | -0.08 | | | 0.00 | | | 0.18 | | | | -0.12 | | | | -0.03 | | | | -0.21 | | | | 0.27 | | | | 0.19 | | | | 0.25 | | | | 0.09 | | | -0.19 | | |  |
| DNER | | -0.39 | | | -0.32 | | | -0.42 | | | 0.24 | | | -0.16 | | | | -0.46 | | | | 0.31 | | | | -0.45 | | | | 0.16 | | | | 0.15 | | | | 0.16 | | | | -0.06 | | | -0.47 | | |  |
| FGF-19 | | -0.02 | | | -0.04 | | | -0.19 | | | 0.08 | | | 0.23 | | | | -0.11 | | | | 0.19 | | | | -0.21 | | | | 0.32 | | | | 0.49 | | | | 0.19 | | | | 0.33 | | | -0.4 | | |  |
| FGF-21 | | -0.18 | | | 0.03 | | | -0.24 | | | 0.00 | | | -0.08 | | | | 0.00 | | | | -0.15 | | | | 0.01 | | | | 0.41 | | | | 0.28 | | | | 0.23 | | | | -0.19 | | | 0.09 | | |  |
|  | | BMI | | | Body fat | | | WHR | | | HbA1c | | | f-Glucose | | | | HOMA | | | | M-value | | | | CRP | | | | Glucagon | | | | Cortisol | | | | ACTH | | | | GH | | | Insulin | | |  |
| **Growth factors** | | | | | | | | | | | | | | | | | | | | | | | | | | | | | | | | | | | | | | | | | | | | | | | | |
| HGF | | -0.20 | | | -0.14 | | | -0.10 | | | -0.12 | | | -0.03 | | | | -0.43 | | | | 0.28 | | | | -0.40 | | | | -0.10 | | | | 0.38 | | | | 0.28 | | | | 0.19 | | | **-0.57** | | |  |
| LAP TGF-β-1 | | -0.17 | | | -0.19 | | | -0.13 | | | 0.33 | | | 0.32 | | | | -0.11 | | | | 0.03 | | | | -0.31 | | | | 0.22 | | | | 0.10 | | | | 0.01 | | | | -0.01 | | | -0.27 | | |  |
| NT-3 | | -0.13 | | | 0.00 | | | -0.29 | | | 0.05 | | | -0.11 | | | | -0.41 | | | | 0.14 | | | | -0.42 | | | | 0.04 | | | | 0.39 | | | | 0.35 | | | | 0.15 | | | **-0.54** | | |  |
| SCF | | -0.29 | | | -0.17 | | | -0.27 | | | -0.08 | | | -0.20 | | | | -0.33 | | | | 0.38 | | | | -0.33 | | | | 0.14 | | | | 0.06 | | | | 0.07 | | | | -0.05 | | | -0.35 | | |  |
| TGF- α | | -0.07 | | | -0.05 | | | 0.09 | | | -0.01 | | | 0.33 | | | | 0.07 | | | | -0.25 | | | | 0.02 | | | | 0.47 | | | | 0.04 | | | | 0.19 | | | | -0.13 | | | 0.04 | | |  |
| VEGF-A | | **-0.66** | | | **-0.5** | | | **-0.51** | | | -0.22 | | | -0.23 | | | | **-0.67** | | | | **0.56** | | | | **-0.69** | | | | -0.16 | | | | 0.14 | | | | 0.24 | | | | -0.17 | | | **-0.63** | | |  |
| **Intracellular signalling molecules** | | | | | | | | | | | | | | |  | | | |  | | | |  | | | | |  | | | |  | | | |  | | | |  | | | |  | | | | |
| 4E-BP1 | | 0.09 | | | -0.01 | | | 0.43 | | | -0.01 | | | 0.14 | | | | 0.16 | | | | -0.22 | | | | -0.07 | | | | 0.07 | | | | 0.14 | | | | -0.1 | | | | -0.19 | | | 0.00 | | |  |
| AXIN1 | | 0.22 | | | 0.26 | | | 0.22 | | | 0.06 | | | 0.37 | | | | 0.21 | | | | -0.29 | | | | 0.02 | | | | 0.24 | | | | 0.30 | | | | 0.1 | | | | -0.02 | | | -0.04 | | |  |
| CASP-8 | | -0.06 | | | -0.13 | | | 0.25 | | | -0.16 | | | 0.09 | | | | 0.04 | | | | -0.03 | | | | -0.22 | | | | -0.03 | | | | 0.17 | | | | 0.03 | | | | -0.19 | | | -0.02 | | |  |
| STAMBP | | 0.07 | | | -0.06 | | | 0.32 | | | -0.03 | | | 0.25 | | | | 0.15 | | | | -0.22 | | | | -0.07 | | | | 0.12 | | | | 0.27 | | | | -0.09 | | | | -0.03 | | | -0.09 | | |  |
| **Receptors** | |  | |  | | |  | | |  | |  | | | |  | | | |  | | | |  | | |  | | | |  | | | |  | | | |  | | | |  | | | |  | |
| CD5 | | -0.22 | | | -0.09 | | | -0.22 | | | -0.15 | | | -0.04 | | | | -0.23 | | | | 0.16 | | | | -0.32 | | | | 0.28 | | | | **0.52** | | | | 0.33 | | | | 0.14 | | | -0.30 | | |  |
| CD6 | | -0.12 | | | 0.04 | | | -0.17 | | | 0.18 | | | 0.03 | | | | -0.20 | | | | -0.04 | | | | -0.24 | | | | 0.37 | | | | 0.32 | | | | 0.47 | | | | 0.16 | | | -0.18 | | |  |
| CD8A | | -0.17 | | | -0.12 | | | -0.22 | | | 0.14 | | | 0.15 | | | | -0.27 | | | | 0.05 | | | | -0.36 | | | | 0.24 | | | | 0.22 | | | | 0.29 | | | | 0.22 | | | -0.38 | | |  |
| CD40 | | -0.16 | | | -0.15 | | | -0.14 | | | 0.27 | | | 0.27 | | | | -0.07 | | | | -0.09 | | | | -0.14 | | | | 0.34 | | | | 0.10 | | | | 0.07 | | | | -0.11 | | | -0.27 | | |  |
| CD244 | | -0.04 | | | 0.04 | | | -0.11 | | | 0.26 | | | 0.22 | | | | 0.00 | | | | -0.21 | | | | -0.18 | | | | **0.54** | | | | 0.39 | | | | 0.31 | | | | 0.08 | | | -0.14 | | |  |
| CDCP1 | | **-0.57** | | | -0.49 | | | **-0.50** | | | -0.13 | | | -0.15 | | | | **-0.57** | | | | 0.37 | | | | **-0.62** | | | | -0.01 | | | | 0.36 | | | | 0.06 | | | | -0.03 | | | **-0.71** | | |  |
| IL-10RB | | -0.32 | | | -0.15 | | | -0.27 | | | -0.32 | | | -0.28 | | | | -0.35 | | | | 0.37 | | | | -0.35 | | | | 0.03 | | | | 0.34 | | | | 0.04 | | | | -0.14 | | | -0.43 | | |  |
| IL-15RA | | 0.08 | | | 0.08 | | | 0.21 | | | -0.30 | | | -0.08 | | | | 0.12 | | | | -0.08 | | | | -0.11 | | | | -0.05 | | | | 0.06 | | | | 0.18 | | | | -0.05 | | | 0.26 | | |  |
| IL-18R1 | | -0.23 | | | -0.18 | | | -0.29 | | | -0.01 | | | 0.13 | | | | -0.29 | | | | 0.16 | | | | -0.35 | | | | 0.32 | | | | 0.36 | | | | 0.27 | | | | 0.26 | | | -0.48 | | |  |
| LIF-R | | -0.09 | | | 0.10 | | | -0.22 | | | 0.22 | | | -0.15 | | | | -0.27 | | | | 0.26 | | | | -0.29 | | | | 0.19 | | | | 0.27 | | | | 0.18 | | | | 0.05 | | | -0.36 | | |  |
| OPG | | -0.15 | | | -0.13 | | | -0.25 | | | 0.27 | | | 0.21 | | | | -0.17 | | | | 0.02 | | | | -0.26 | | | | 0.34 | | | | 0.12 | | | | 0.31 | | | | 0.20 | | | -0.19 | | |  |
| PD-L1 | | -0.19 | | | -0.05 | | | -0.40 | | | 0.18 | | | 0.11 | | | | -0.28 | | | | 0.16 | | | | -0.16 | | | | 0.45 | | | | 0.43 | | | | 0.43 | | | | 0.38 | | | -0.42 | | |  |
| SLAMF1 | | -0.43 | | | **-0.58** | | | -0.02 | | | -0.34 | | | -0.02 | | | | -0.27 | | | | 0.03 | | | | -0.49 | | | | -0.31 | | | | 0.04 | | | | -0.31 | | | | -0.15 | | | -0.42 | | |  |
| TRAIL | | -0.11 | | | -0.02 | | | -0.07 | | | -0.05 | | | 0.04 | | | | -0.17 | | | | -0.02 | | | | -0.31 | | | | 0.27 | | | | 0.42 | | | | 0.33 | | | | 0.20 | | | -0.26 | | |  |

Inflammatory responses are $\Delta$ with respect to fasting levels. Hormonal responses are $\Delta$AUC or AUC (insulin) of hyperglycemic phase (30-165 min of hyperglycemic clamp). Body fat= in % as obtained by bioimpedance, WHR= Waist-hip ratio, f-Glucose= fasting plasma glucose, GH= growth hormone. All metabolic parameters were obtained at first occasion. Background in three color scale where blue refers to negative coefficients and red refers to positive coefficients, increasing intensity with higher magnitude. White refers to a coefficient of 0. Significant coefficients are typed in **bold**.
